# Advanced Multi-Level Ensemble Learning Approaches for Comprehensive Sperm Morphology Assessment

**DOI:** 10.3390/diagnostics15121564

**Published:** 2025-06-19

**Authors:** Abdulsamet Aktas, Taha Cap, Gorkem Serbes, Hamza Osman Ilhan, Hakkı Uzun

**Affiliations:** 1 Department of Computer Engineering, Faculty of Technology, Marmara University, 34840 Istanbul, Turkey; abdulsamet.aktas@marmara.edu.tr; 2 Department of Control and Automation Engineering, Faculty of Electrical and Electronics, Yildiz Technical University, 34220 Istanbul, Turkey; taha.cap@std.yildiz.edu.tr; 3 Department of Biomedical Engineering, Faculty of Electrical and Electronics, Yildiz Technical University, 34220 Istanbul, Turkey; gserbes@yildiz.edu.tr; 4 Department of Computer Engineering, Faculty of Electrical and Electronics, Yildiz Technical University, 34220 Istanbul, Turkey; hoilhan@yildiz.edu.tr; 5 Department of Urology, Faculty of Medicine, Recep Tayyip Erdoğan University, 53020 Rize, Turkey

**Keywords:** feature extraction, penultimate layer classification, combined decision mechanisms, sperm morphology, Support Vector Machines

## Abstract

**Introduction:** Fertility is fundamental to human well-being, significantly impacting both individual lives and societal development. In particular, sperm morphology—referring to the shape, size, and structural integrity of sperm cells—is a key indicator in diagnosing male infertility and selecting viable sperm in assisted reproductive technologies such as in vitro fertilisation (IVF) and intracytoplasmic sperm injection (ICSI). However, traditional manual evaluation methods are highly subjective and inconsistent, creating a need for standardized, automated systems. **Objectives:** This study aims to develop a robust and fully automated sperm morphology classification framework capable of accurately identifying a wide range of morphological abnormalities, thereby minimizing observer variability and improving diagnostic support in reproductive healthcare. **Methods:** We propose a novel ensemble-based classification approach that combines convolutional neural network (CNN)-derived features using both feature-level and decision-level fusion techniques. Features extracted from multiple EfficientNetV2 variants are fused and classified using Support Vector Machines (SVM), Random Forest (RF), and Multi-Layer Perceptron with Attention (MLP-Attention). Decision-level fusion is achieved via soft voting to enhance robustness and accuracy. **Results:** The proposed ensemble framework was evaluated using the Hi-LabSpermMorpho dataset, which contains 18 distinct sperm morphology classes. The fusion-based model achieved an accuracy of 67.70%, significantly outperforming individual classifiers. The integration of multiple CNN architectures and ensemble techniques effectively mitigated class imbalance and enhanced the generalizability of the model. **Conclusions:** The presented methodology demonstrates a substantial improvement over traditional and single-model approaches in automated sperm morphology classification. By leveraging ensemble learning and multi-level fusion, the model provides a reliable and scalable solution for clinical decision-making in male fertility assessment.

## 1. Introduction

Sperm morphology assessment plays a crucial role in diagnosing male infertility, as abnormalities in sperm shape, size, and structure can indicate underlying reproductive pathologies. Historically, infertility has been a documented concern for millennia, with references dating back 4000 years to Assyrian marriage contracts [1]. Today, infertility affects approximately 17.5% of adults globally, defined by the World Health Organization (WHO) as the inability to conceive after 12 months of regular unprotected intercourse [2]. Semen analysis, focusing on parameters such as sperm count, motility, and particularly morphology, remains essential for evaluating male fertility potential and determining appropriate infertility treatments [3,4].

Traditionally, sperm morphology evaluation has relied on manual microscopic examination, which is labor-intensive, subjective, and heavily dependent on the evaluator’s expertise, resulting in significant inter-observer variability [5,6]. To address these challenges, computer-assisted sperm analysis (CASA) systems have been developed, providing faster and less subjective results. However, CASA systems have limitations including high costs, integration difficulties, and a primary focus on motility rather than detailed morphological abnormalities, often yielding inconsistent morphological evaluations [7,8]. Therefore, there is a pressing need for robust, fully automated sperm morphology analysis systems capable of accurately and consistently identifying diverse morphological abnormalities.

In line with the critical clinical role of sperm morphology assessment, recent breakthroughs in computational science have transformed traditional evaluation methodologies. State-of-the-art techniques, particularly those grounded in machine learning (ML) and deep learning (DL), now enable the automation of intricate processes such as sperm component segmentation, discriminative feature extraction, and morphological classification [9,10,11,12,13,14,15]. Among these, convolutional neural networks (CNNs) have emerged as a dominant paradigm, exhibiting exceptional efficacy in biomedical image analysis, including the high-precision categorization of sperm morphology. By minimizing inter-observer variability and enhancing analytical scalability, these data-driven approaches provide standardized, objective, and reproducible diagnostic outcomes. Building upon these advancements, our study introduces a novel, end-to-end automated classification framework that integrates multi-level feature fusion with optimized machine learning classifiers, thereby improving diagnostic accuracy and reinforcing clinical decision-support systems.

Recent advances in machine learning, particularly deep learning techniques such as CNNs, have significantly improved the accuracy of sperm morphology analysis by automating feature extraction processes [16,17]. However, despite these improvements, gaps remain. Existing deep learning-based methods frequently focus solely on head morphology and neglect the comprehensive segmentation and classification of other critical sperm components, such as the mid-piece and tail. Furthermore, these methods often lack interpretability and require large, diverse datasets for training, which are currently limited.

Our study addresses these limitations by leveraging CNN-derived features through advanced fusion techniques to enhance classification performance. By combining features extracted from multiple CNN models and utilizing both feature-level and decision-level fusion, we aim to exploit complementary strengths from different feature representations. Specifically, we applied Support Vector Machines (SVMs) [18], Random Forest (RF) [19], and Multi-Layer Perceptron with attention mechanisms (MLP-A) to enhance classification robustness [20]. The effectiveness of these fusion strategies is evaluated using our recently proposed comprehensive dataset, “Hi-LabSpermMorpho” [21], designed to include diverse abnormalities with a balanced representation across various morphological classes. Through the integration of fusion techniques and a comprehensive dataset, this study seeks to establish a robust, automated system for sperm morphology analysis, significantly improving diagnostic accuracy and supporting better clinical decision-making for infertility treatments.

Manual sperm morphology analysis is time-consuming, subjective, and highly dependent on expert evaluation, often resulting in inter-observer variability [5,6]. Consequently, there is increasing demand for automated, accurate, and robust sperm morphology assessment systems.

Initial approaches to sperm morphology classification employed traditional machine learning methods with manual feature extraction. Alegre et al. utilized contour features extracted via Otsu thresholding, achieving a notably low error rate of 1% [22]. They further demonstrated the effectiveness of texture-based features, obtaining 94% accuracy using Multilayer Perceptron (MLP) and KNN classifiers [23]. Ilhan et al. applied wavelet transforms to extract features from sperm images, reporting improved classification accuracy due to better directional selectivity and shift invariance of Dual Tree Complex Wavelet Transform (DTCWT) [14]. In subsequent research, descriptor-based features (KAZE, SURF, and MSER) were combined with wavelet features for classification through traditional and ensemble learning methods, achieving high accuracy values [24].

The advent of deep learning, particularly CNNs, has significantly enhanced automated feature extraction capabilities in sperm morphology analysis [10]. Nissen et al. proposed a CNN-based method demonstrating superior performance over classical image analysis, achieving precision and recall values of approximately 93.87% and 91.89%, respectively [25]. Movahed et al. developed a hybrid approach integrating CNNs for sperm head segmentation with traditional classifiers for tail and mid-piece detection, achieving a Dice coefficient of 0.90 for head segmentation [26].

In the field of sperm morphology classification, several studies have demonstrated the effectiveness of ensemble methods that combine multiple CNN architectures. For example, Spencer et al. integrated VGG16, DenseNet-161, and a modified ResNet-34 with a meta-classifier, achieving an F1 score of 98.2% on the HuSHeM dataset [12]. Similarly, Yuzkat et al. employed ensemble learning by combining multiple CNN models, attaining high classification accuracies across various datasets [15]. Ilhan et al. enhanced performance further by integrating voting mechanisms between VGG16 and GoogleNet, resulting in significant accuracy improvements [27]. Lightweight CNN models have also been explored in this domain, with Iqbal et al. demonstrating effective sperm head morphology classification with minimal computational complexity [28].

When considering hybrid approaches that combine deep feature extraction with machine learning classifiers, notable advancements have been made in medical image classification. Salama et al., for example, proposed a hybrid framework for COVID-19 detection by extracting optimal layer features from ten different deep CNN models and classifying them with five distinct machine learning classifiers such as SVM and Random Forest, achieving a high accuracy of 99.39% [29]. Similarly, Verma et al. developed a deep feature extraction and ensemble learning-based framework for multi-class classification of retinal fundus images, targeting diseases like diabetic retinopathy and macular degeneration. Their approach, leveraging models such as NASNetMobile, VGG16, and DenseNet for feature extraction combined with Random Forest, Extra Trees, and Histogram Gradient Boosting classifiers, achieved an accuracy of 87.2% and an F1-score up to 99%, outperforming previous methods [30]. Likewise, Çelik et al. introduced a hybrid classification model for brain tumor detection in MRI images, combining a novel CNN-based feature extractor with optimized machine learning classifiers using Bayesian optimization for hyperparameter tuning. Their method achieved a top classification accuracy of 97.15% and demonstrated superior performance and efficiency compared to other CNN classifiers and hybrid models [31].

Beyond these, in other medical imaging applications, Mabrouk et al. developed an ensemble learning-based computer-aided diagnosis system for pneumonia detection on chest X-ray images. By fine-tuning three pretrained CNN architectures—DenseNet169, MobileNetV2, and Vision Transformer—and fusing their extracted features, the model achieved 93.91% accuracy and a 93.88% F1-score, surpassing previous state-of-the-art approaches [32]. Additionally, Zhang et al. proposed a novel deep learning framework for sperm head morphology classification that enhances robustness by incorporating anatomical and image priors through pseudo-mask generation and unsupervised spatial prediction tasks. Their method achieved state-of-the-art performance on two public datasets, with 65.9% accuracy on SCIAN and 96.5% on HuSHeM, effectively handling noisy labels without requiring extra manual annotation [33].

Despite these advancements, several gaps still persist. Existing datasets such as HuSHeM, SCIAN-SpermMorphoGS, and SMIDS are limited by their relatively small size and restricted number of morphological classes, hindering the development of comprehensive systems [7,16,24]. Thus, there remains a significant need for larger, more diverse datasets and innovative fusion strategies to address these limitations and further enhance the accuracy and robustness of automated sperm morphology classification systems.

This research provides a comprehensive review of existing sperm morphology analysis methods, emphasizing the advancements brought by ensemble and fusion learning strategies. Despite significant progress, limitations remain concerning classification robustness and generalizability, particularly when utilizing CNN-based features independently. Our current study addresses these limitations by

- Implementing feature-level fusion by combining features extracted from multiple EfficientNetV2 models to leverage complementary strengths and enhance classification accuracy.
- Investigating decision-level fusion strategies, specifically employing soft voting across multiple classifiers (SVM, RF, and MLP-A), to improve overall model robustness.
- Evaluating the impact of dimensionality reduction via dense-layer feature transformations on classification accuracy and computational efficiency, thus highlighting practical approaches to optimize model performance.
- Performing detailed analysis on the effects of ensemble learning methods for low-sample classes, providing insights into how fusion techniques can address data imbalance issues prevalent in clinical datasets.
- Conducting extensive experimentation using the Hi-LabSpermMorpho dataset, which includes 18 distinct sperm morphology classes and 18,456 image samples, to ensure the broad applicability and validity of the proposed fusion-based approaches.
- Emphasizing the intended usability of the proposed system by clinical professionals and diagnostic laboratories as a decision-support tool for automated sperm morphology analysis in routine testing environments.

## 2. Materials and Methods

### 2.1. Dataset Information

**Figure 1:**
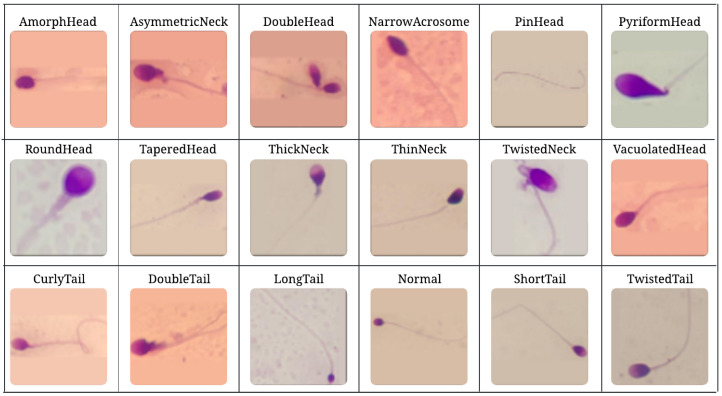
Sample sperm images from the Hi-LabSpermMorpho dataset [21], illustrating representative examples from each of the 18 morphological classes, including head, neck, and tail abnormalities, as well as the Normal class.

The Hi-LabSpermMorpho [21] is a comprehensive sperm morphology dataset designed specifically for developing automated sperm morphology classification systems. It provides the detailed labeling of various sperm abnormalities observed in the head, neck (mid-piece), and tail regions, as well as normal sperm morphology. The dataset includes 18 distinct classes, namely: AmorphHead, AsymmetricNeck, CurlyTail, DoubleHead, DoubleTail, LongTail, NarrowAcrosome, Normal, PinHead, PyriformHead, RoundHead, ShortTail, TaperedHead, ThickNeck, ThinNeck, TwistedNeck, TwistedTail, and VacuolatedHead. Apart from the “Normal” class, all other classes represent abnormal sperm morphologies, which can be further grouped into four superclasses: Normal, Head Abnormalities, Tail Abnormalities, and Neck Abnormalities. An overview of the 18 sperm morphology classes included in the Hi-LabSpermMorpho dataset is presented in Figure 1, demonstrating the visual diversity and structural distinctions among normal and abnormal sperm samples.

The dataset was developed according to the WHO criteria for sperm morphology analysis [34]. To effectively highlight morphological abnormalities, a Diff-Quick staining kit (BesLab) was used. This staining kit includes a fixative solution with triarylmethane stain dissolved in methanol, an eosinophilic xanthene staining solution (reagent 1), and a basophilic thiazine staining solution (reagent 2), each with varying dosages leading to different color intensities. During sample preparation, air-dried sperm smears were sequentially immersed in the fixative solution and staining reagents, each immersion followed by draining excess solution vertically on absorbent paper. Finally, the slides were washed under running water and allowed to dry, after which immersion oil was applied for clear visualization.

Images were acquired using ZEISS AXIO LAB A1 and Olympus BX43 microscopes under bright-field microscopy at 100× magnification. A custom-designed mobile phone-mounted apparatus integrated into the microscope’s ocular facilitated image capturing. For the BesLab staining kit, the Hi-LabSpermMorpho dataset contains 18,456 annotated RGB images categorized into 18 classes, providing one of the most extensive resources for sperm morphology analysis. Table 1 summarizes the detailed distribution of samples across all morphological classes, highlighting a significant class imbalance that poses challenges for model training and evaluation. The dataset’s diversity and detailed labeling significantly facilitate the development of robust, generalizable automated systems capable of accurately classifying various sperm abnormalities.

The dataset supports robust evaluation methods, such as K-fold cross-validation (K = 5), ensuring comprehensive utilization and reliable model generalization, especially critical given the imbalanced nature of the dataset.

### 2.2. The Proposed Approach

**Figure 2:**
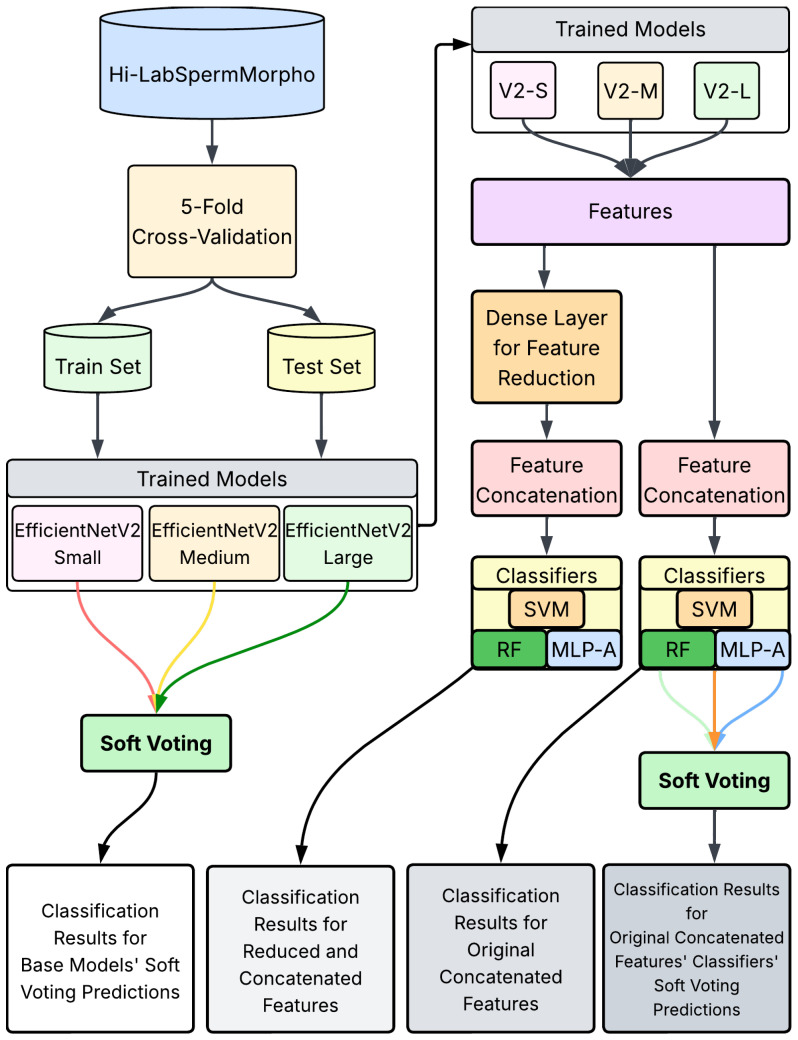
An overview of the proposed classification pipeline. The diagram illustrates the full workflow, using trained EfficientNetV2 models.

The proposed approach aims to enhance sperm morphology classification performance by leveraging both feature-level and decision-level fusion techniques applied to features extracted from CNN-based models, as shown in Figure 2. To overcome the limitations observed in individual CNN models, particularly regarding lower classification accuracies and limited generalization capabilities, a systematic fusion framework consisting of multiple steps was developed. This framework integrates various feature extraction, reduction, concatenation, and fusion methodologies to optimize classification outcomes.

Firstly, the EfficientNetV2 architectures—Small (S), Medium (M), and Large (L)—were individually trained on the Hi-LabSpermMorpho dataset. These architectures are known for their compound scaling strategy, which balances network depth, width, and resolution to achieve improved accuracy and efficiency. EfficientNetV2-S offers a lightweight and fast model suitable for lower-resource settings, while EfficientNetV2-M and EfficientNetV2-L provide increasingly deeper and wider networks capable of capturing more complex representations, which is especially beneficial for distinguishing among the subtle morphological variations in sperm cells.

After training, deep features were extracted from the penultimate (second-to-last) fully connected layer of each CNN model. Extracting features from the penultimate layer provides a high-level abstraction of sperm morphological characteristics, capturing both global and local visual attributes essential for distinguishing between sperm morphology classes.

In the second step, due to the high dimensionality of the extracted feature sets, dimensionality reduction was applied using a dense (fully connected) layer to produce compact yet informative feature representations. This step ensures that irrelevant or redundant information within the high-dimensional CNN-extracted features is minimized, allowing classifiers to more effectively utilize the reduced features in subsequent stages.

Next, the reduced feature vectors from each of the three CNN architectures were concatenated to create an integrated feature representation. By combining complementary information from EfficientNetV2-Small, EfficientNetV2-Medium, and EfficientNetV2-Large models, the resulting concatenated feature vector offers enhanced representational capacity compared to features derived from any single model. This concatenation step significantly enriches the discriminatory power of the integrated feature set, thus improving the potential performance of the classifier algorithms.

In the fourth step, the individual classification was performed by training classical machine learning classifiers—including SVMs, RF, and MLP-A—using the concatenated feature vectors. Each classifier independently learned to discriminate among sperm morphology classes based on the fused feature representations, leveraging distinct decision boundaries and learning mechanisms. Finally, decision-level fusion was applied to aggregate the outputs of the various classifiers. Employing ensemble learning strategies such as soft voting, the final classification decision was determined.

The selection of these classifiers is grounded in their complementary strengths and established effectiveness in complex multi-class problems. SVMs are particularly suited for handling high-dimensional data with closely related classes due to their ability to maximize margins between decision boundaries, which is critical for distinguishing subtle morphological differences. RF provides robustness against overfitting and efficiently manages class imbalance by averaging multiple decision trees. MLP-A captures complex, non-linear patterns in data and emphasizes relevant features through attention mechanisms. This combination leverages diverse decision-making strategies, improving classification performance, as also supported by the extensive literature where CNN-derived features are combined with classical classifiers like SVM for enhanced accuracy in medical image analysis [29,35,36].

This combined feature-level and decision-level fusion strategy harnesses the complementary strengths of multiple CNN-derived feature sets and diverse classifier outputs, significantly enhancing the final classification accuracy. By systematically integrating these steps, the proposed method addresses the challenges inherent in sperm morphology classification, including high intra-class variability, limited dataset sizes, and class imbalance issues. Overall, the proposed fusion-based framework demonstrates improved generalization and higher accuracy compared to individual CNN models, establishing a robust, comprehensive, and reliable approach for automated sperm morphology analysis. The details of the proposed approach are given in subsections.

#### 2.2.1. Feature Concatenation and Reduction

To enhance classification performance, we explore feature concatenation strategies that integrate different scales of extracted feature representations. The concatenation is performed along the feature dimension, effectively creating an augmented feature representation that provides a broader and more diverse set of discriminative attributes for the classifier.

**Figure 3:**
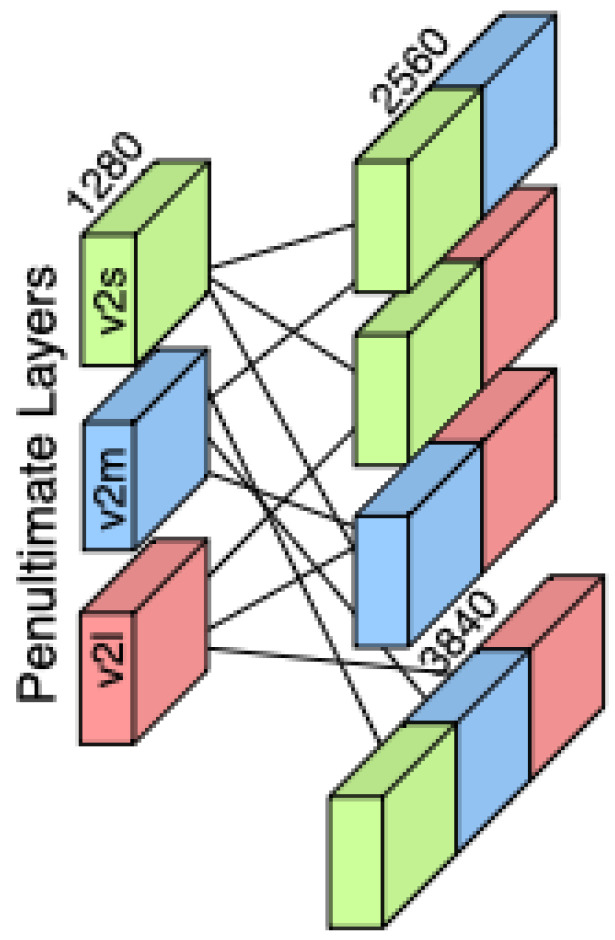
An illustration of the feature concatenation strategy. Feature vectors extracted from the penultimate layers of EfficientNetV2-S (green), V2-M (blue), and V2-L (red) are combined in pairwise and triple-wise configurations.

As illustrated in Figure 3, the feature concatenation process combines the outputs of three EfficientNetV2 variants—S, M, and L—by aligning their penultimate layer feature vectors along the feature dimension. This fusion enriches the representation space by capturing complementary semantic information at multiple network depths, supporting more robust downstream classification. The feature vectors are extracted from the penultimate layer of each EfficientNetV2 variant (S, M, and L), preserving semantic richness while avoiding task-specific transformations. Denoting these vectors by $F_{S},F_{M},F_{L}\in R^{1280}$, we create variances of combinations as given before and concatenate them along their feature dimension:

(1) $$F_{S},F_{M},F_{L}\in R^{1280},$$

we create both double-fusion and triple-fusion representations by concatenating them along their feature dimensions. Specifically, for the double-fusion representations, we have

(2) $$\left[F_{S};F_{M}\right],\;\left[F_{S};F_{L}\right],\;\left[F_{M};F_{L}\right]\;\in \;R^{2560},$$

while the triple-fusion representation is given by

(3) $$\left[F_{S};F_{M};F_{L}\right]\;\in \;R^{3840}.$$

**Figure 4:**
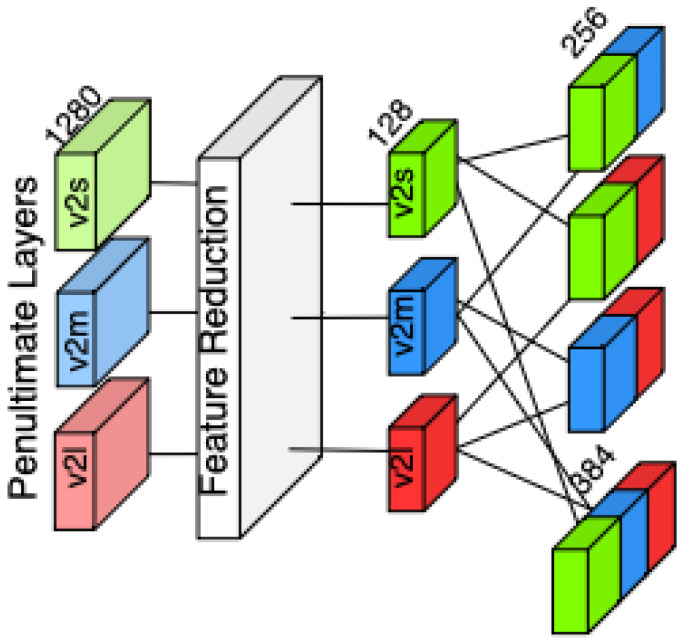
Illustration of the feature concatenation strategy. Feature vectors extracted from the dense layer after penultimate layers’ features of EfficientNetV2-S (bright green), V2-M (bright blue), and V2-L (bright red) are combined in pairwise and triple-wise configurations.

Also, we created feature variants using a dense layer right after the penultimate layer, creating a variant with 128 features as depicted in Figure 4. We then concatenated these features with the same approach just as we did with penultimate layer outputs, to measure if the feature vector size makes any difference in the performance of classifiers.

The extracted 1280-dimensional feature representation is projected into a lower-dimensional latent space of 128 features using a fully connected transformation, followed by a non-linear activation function. This step aims to understand the representation power differences between the original penultimate layer and reduced-size penultimate layer, while smaller feature sizes makes classifiers works faster. This dense layer that reduces features applies a Rectified Linear Unit (ReLU) activation, ensuring the preservation of discriminative properties while enhancing the model’s ability to capture relevant patterns. By performing this dimensionality reduction, we aim to evaluate whether the transformed feature representation improves the classification performance and whether feature size impacts the robustness of the classifiers. With these feature variations, we obtain eight sets of features.

#### 2.2.2. Feature Classifiers

##### SVM

Support Vector Machines (SVMs) were selected in this study due to their robustness in handling high-dimensional feature vectors derived from deep convolutional models. In the context of the classification of sperm morphology, where numerous categories exhibit subtle structural differences, the margin-based learning principle of SVM offers an effective way to distinguish between closely related patterns.

Among the kernel functions evaluated, the Radial Basis Function (RBF) kernel demonstrated superior performance. This can be attributed to its ability to map the input features into a higher-dimensional space, which helps in separating classes that are not linearly separable in the original feature space. Such flexibility is particularly valuable in our application, where concatenated features from multiple CNN models result in complex and overlapping distributions.

Overall, SVM’s capacity to construct flexible decision boundaries without relying heavily on large sample sizes makes it well-suited for medical image analysis tasks like our 18-class problem, where data imbalance and subtle inter-class variations are common [37].

The formulation of the RBF kernel is given by

(4) $$K\left(x_{i},x_{j}\right)=\exp\left(-\gamma ∥x_{i}-x_{j}{∥}^{2}\right),$$

where γ is a hyperparameter controlling the influence of individual training samples. Properly tuning γ ensures the model captures both local structures and global class distributions, thereby reducing misclassification rates.

The SVM training process begins with hyperparameter tuning, focusing on the kernel coefficient (γ) and the regularization parameter (*C*). We used cross-validation to find values that balance underfitting and overfitting, ensuring robust generalization. Before training on the concatenated features, we applied standardization so that each dimension contributes equally, preventing any feature from disproportionately influencing the decision boundary. To handle multi-class classification, we adopted a one-vs-rest strategy: a separate binary classifier is trained for each class against the rest, and final predictions are made by selecting the classifier with the highest confidence score. This approach effectively handles our 18-class setup and leverages the rich information provided by the concatenated feature vectors.

##### MLP-Attention

Feature selection is crucial in high-dimensional feature spaces to emphasize relevant information while reducing noise [38]. To achieve this, we incorporate an MLP attention module applied on the concatenated CNN-based feature vectors. This module assigns higher weights to class-discriminative features before classification, effectively highlighting important information and suppressing irrelevant or noisy features [39].

The MLP attention module adapts dynamically during training, which improves the model’s ability to generalize to unseen samples. Structurally, it consists of a fully connected layer followed by a sigmoid activation function that learns feature-wise attention scores. These scores are then used to weight the input features adaptively before they are passed to the classifier.

This approach enables more effective discrimination in our challenging 18-class sperm morphology classification problem, where subtle differences between classes exist. By leveraging complementary information from multiple CNN architectures rather than relying on individual models alone, the module enriches the representation space with more meaningful and discriminative features.

(5) $$A=\sigma \left(W_{\mathrm{attn}}F+b_{\mathrm{attn}}\right),$$

where $W_{\mathrm{attn}}\in R^{d\times d}$ is the learnable weight matrix, $b_{\mathrm{attn}}\in R^{d}$ is the bias term, σ(·) is the sigmoid activation function, and $F\in R^{d}$ represents the input feature vector. The resulting attention weights $A\in R^{d}$ are applied to the input features via element-wise multiplication:

(6) $$F_{\mathrm{attended}}=A⊙F,$$

where ⊙ denotes element-wise multiplication. This operation enhances the most relevant features and suppresses less informative ones. After feature refinement using the attention mechanism, the classifier projects the modified feature vector $F_{\mathrm{attended}}$ into the final output space using a linear transformation:

(7) $$Y=W_{\mathrm{final}}F_{\mathrm{attended}}+b_{\mathrm{final}},$$

where $W_{\mathrm{final}}\in R^{18\times d}$ and $b_{\mathrm{final}}\in R^{18}$ are learnable parameters, and $Y\in R^{18}$ represents the unnormalized logits. The softmax function converts the logits into class probabilities:

(8) $$P\left(y_{i}|x\right)=\frac{\exp(Y_{i})}{\sum_{j=1}^{18}\exp\left(Y_{j}\right)}.$$

and the predicted class is chosen by selecting the index *i* with the highest probability.

##### Random Forest Classifier

In the context of sperm morphology classification, where high-dimensional and complex feature spaces are derived from multiple CNN models, effective feature selection and robust classification methods are critical. RF is an ensemble learning algorithm that constructs a multitude of decision trees using random subsets of features and training samples, making it particularly suited for handling noisy and heterogeneous biomedical data [19].

RF improves the classification of subtle morphological differences among sperm cells by aggregating diverse tree-based predictions, thereby reducing the risk of overfitting to specific feature patterns that might occur in single models. This ensemble approach provides resilience to noisy or redundant features frequently encountered in clinical datasets. Additionally, RF’s inherent mechanism of random feature selection during tree construction helps in identifying the most informative features relevant to distinguishing between normal and abnormal sperm morphologies.

The majority voting scheme used in RF combines predictions from individual trees to deliver stable and robust classification results across the challenging 18-class sperm morphology dataset, which contains closely related subtypes with fine-grained variations.

To maintain consistency and improve the learning process across features obtained at different scales and from diverse CNN architectures, feature standardization is applied:

(9) $$F_{\mathrm{scaled}}=\frac{F-\mu }{\sigma },$$

where *F* represents the raw feature matrix, μ is the mean, and σ is the standard deviation of each feature. This transformation ensures that all input dimensions contribute equally to the classification process. The RF classifier then learns an ensemble of *T* decision trees, where each tree produces a probability estimate for a given class. The final class probabilities are computed as

(10) $$P\left(y|x\right)=\frac{1}{T}\sum_{t=1}^{T}P_{t}\left(y|x\right),$$

where $P_{t}\left(y|x\right)$ represents the predicted probability from the *t*-th decision tree. The final class prediction is determined by selecting the class with the highest probability:

(11) $$\hat{y}=\arg\;\max_{y}P\left(y|x\right).$$

##### Soft Voting-Based Decision Level Fusion

Soft voting is an ensemble method that integrates the outputs of multiple predictive models to yield a more robust decision. In this approach, each model independently produces a set of class probabilities—often stored in a comma-separated value (CSV) format—which represent its confidence in each possible class label. These probability estimates are then aggregated, typically by averaging, to generate a consensus decision. Because soft voting harnesses the collective strengths of multiple models, it can effectively dilute the impact of weaker individual predictions, leading to reduced overall error and improved predictive accuracy.

**Figure 5:**
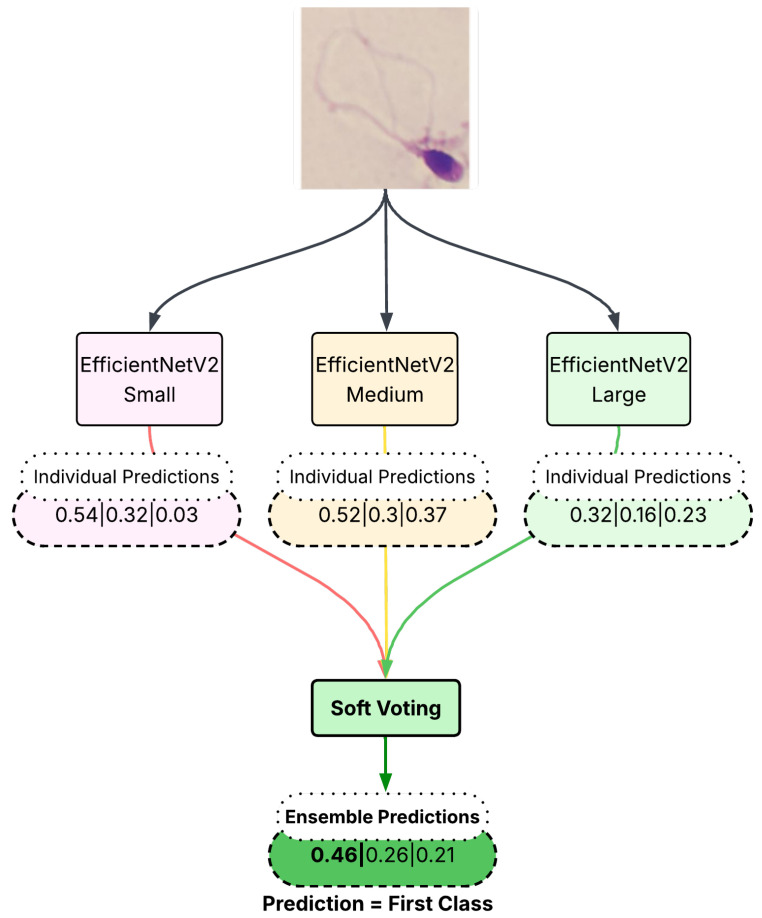
Demonstration of soft voting. Each classifier contributes a probability distribution, and the final class is determined based on the highest cumulative probability.

This approach considers the predicted class probabilities obtained from the softmax layer of each individual network. Instead of relying solely on the highest probability, soft voting takes the average of predicted probabilities across multiple networks and selects the class with the highest cumulative probability. This method ensures that misclassified samples in individual networks have a reduced negative impact on the final decision. When there are contradictory classifications, probability-based fusion allows the correctly classified networks to have a greater influence in the decision-making process, ultimately improving classification accuracy. The visual explanation of the employed soft voting approach is given in Figure 5.

Formally, for a given test sample *x*, let $p_{k}\left(y|x\right)$ denote the probability assigned to class *y* by classifier *k*. The final decision under soft voting is given by

(12) $$y^{*}=\arg\max_{y}\sum_{k=1}^{K}p_{k}\left(y|x\right)$$

## 3. Results

### 3.1. Experimental Setup

All experiments were conducted using the PyTorch 2.5.1 deep learning framework and Scikit-Learn on a workstation equipped with NVIDIA RTX 4050 6 GB (NVIDIA Corporation, Santa Clara, CA, USA), 13th Gen Intel Core i5-13500H (Intel Corporation, Santa Clara, CA, USA), and 16 GB Kingston RAM (Kingston Technology Corporation, Fountain Valley, CA, USA). The operating system used was Ubuntu 24.04.2 LTS (Canonical Ltd., London, UK), and model training was accelerated using CUDA support. Some of classifier trainings are accelerated on a CPU unit. Each model was trained and evaluated using 5-fold cross-validation on the Hi-LabSpermMorpho dataset. Reported accuracies represent the average performance across all folds, providing a reliable estimate under data imbalance conditions.

### 3.2. Individual Learning Results

Evaluating individual CNN models provides insight into their standalone performance before analyzing the enhancements brought by ensemble approaches. As highlighted earlier, our dataset presents significant class imbalance, posing a challenge for individual CNN architectures. Table 2 presents the classification accuracies achieved by EfficientNetV2 variants, each trained individually (with the original features where feature reduction was not applied) using different optimizers as also published in [21]. In reference to this baseline study [21], it was determined that the peak accuracies were achieved using EfficientNet v2 models. Consequently, Multi-Level Ensemble Learning Strategies were developed and implemented utilizing these particular models to enhance performance further.

The results show relatively moderate performance for individual models, primarily due to the dataset’s inherent complexity and imbalanced class distributions. To better understand the potential for improvement, we explored different kernel functions using SVMs applied individually to the feature sets extracted by each EfficientNetV2 variant. Table 3 summarizes these results.

Consistently, the RBF kernel demonstrated superior accuracy across all EfficientNetV2 variants. While polynomial and linear kernels also provided competitive outcomes, their limitations in handling nonlinear class boundaries restricted their overall effectiveness. The Sigmoid kernel showed the lowest performance due to instability issues in high-dimensional feature spaces and sensitivity to parameter tuning.

Though individual CNN models provide a valuable baseline performance, their accuracies highlight a substantial opportunity for improvement. The subsequent sections explore the performance enhancements obtained through ensemble learning techniques, combining complementary features from these CNN models to create a more robust classification framework.

### 3.3. Classification Results for Base Models’ Soft Voting Predictions

To assess the performance gains of ensemble decision-making, we implemented a soft voting strategy. This method aggregates the predicted class probabilities from multiple classifiers trained on different EfficientNetV2 variants (trained with the original features where feature reduction was not applied) and selects the final class based on the highest combined confidence. Table 4 summarizes the classification accuracies obtained using different combinations of EfficientNetV2 models in the soft voting ensemble. These combinations include two-model and three-model configurations.

The results indicate that incorporating multiple views of feature representations via soft voting consistently improves classification performance. In particular, the three-model combination achieved the highest accuracy, suggesting that increased feature diversity and probabilistic consensus contribute to a more robust decision-making process. Additionally, increasing density difference, such as combining small and large model variants, makes probabilities improves probability distribution across different feature spaces.

### 3.4. Classification Results for Original Concatenated Features

To evaluate the effectiveness of different classifiers on combined feature representations, we conducted experiments using concatenated features from EfficientNetV2 models: V2-S + V2-M, V2-S + V2-L, V2-M + V2-L, and V2-S + V2-M + V2-L. Each concatenated feature vector was used as input to three classifiers: SVM, MLP-A, and RF. Table 5 presents the classification accuracies achieved by each classifier for the respective feature combinations.

The results demonstrate that feature concatenation improves classifier performance across all models. Among the classifiers, RF achieved the highest overall accuracy with the EfficientNetV2-S + V2-M + V2L configuration. These findings highlight the advantage of integrating complementary feature representations and attention mechanisms for more robust classification in sperm morphology analysis. It is also noticed that classifiers for combined features get higher accuracies than base model classification performances.

### 3.5. Classification Results for Reduced and Concatenated Features

One of the primary challenges in computational modeling, especially in combined classification approaches, is ensuring time efficiency while managing high-dimensional data. Reducing feature dimensionality not only enhances computational speed but also mitigates the “curse of dimensionality”, a common issue where an excessive number of features negatively impacts model performance by causing data sparsity, increased computational complexity, and overfitting. Effective feature reduction aims to decrease dimensionality while retaining the most informative aspects of the data.

To address these issues, dimensionality reduction techniques, such as applying a dense layer, are employed. This method reduces the feature space size while aiming to retain the essential characteristics and valuable information contained within the original features, thereby enhancing classification efficiency and generalizability.

The classification accuracies obtained by different classifiers using the reduced and concatenated feature combinations are presented in Table 6.

**Figure 6:**
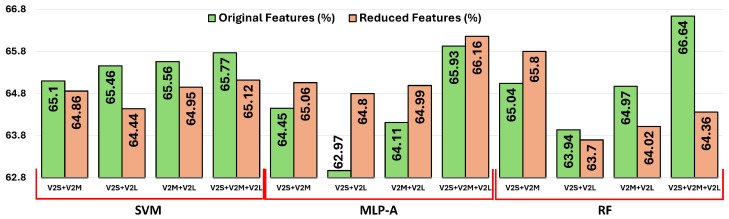
A comparison of classification accuracies obtained using both original and reduced concatenated features across the three classifiers (SVM, MLP-A, and RF) and four different CNN feature fusion combinations: (i) EfficientNetV2-S + V2-M, (ii) EfficientNetV2-S + V2-L, (iii) EfficientNetV2-M + V2-L, and (iv) EfficientNetV2-S + V2-M + V2-L. Each group of bars corresponds to one fusion configuration, and within each group, the classification results using the three classifiers are presented. Light red bars indicate the results obtained using the original high-dimensional concatenated features, while light green bars represent the results after applying our proposed feature reduction method. This figure illustrates the effect of both feature fusion and our custom reduction technique on overall classification performance.

As shown in Figure 6, the results obtained from the original and reduced feature sets highlight classifier-specific behaviors concerning dimensionality reduction. MLP-A consistently benefits from reduced features, likely due to its attention mechanism, which selectively emphasizes informative patterns and suppresses noise. This enhancement helps mitigate the negative impacts of dimensionality, such as redundancy and irrelevant features. RF shows stable performance across both configurations, slightly benefiting from dimensionality reduction, as ensemble methods inherently handle high-dimensional data well but still gain marginal efficiency and robustness improvements when redundant information is eliminated. Conversely, SVM tends to perform marginally better with higher-dimensional, information-rich inputs, as its margin-based optimization approach leverages subtle feature distinctions to identify class boundaries. Consequently, SVM experiences a slight performance drop when features are reduced.

Overall, employing feature reduction via dense layers provides computational efficiency and effectively addresses the curse of dimensionality, thus achieving minimal loss in performance. The benefits, however, vary depending on each classifier’s sensitivity to input dimensionality.

### 3.6. Classification Results for Original Concatenated Features’ Classifiers’ Soft Voting Predictions

To improve overall classification robustness, we implemented decision-level fusion using soft voting across multiple classifiers trained on concatenated 1280-dimensional feature vectors. This approach integrates the probability outputs of SVM, MLP-A, and RF classifiers for each test sample. By averaging class probabilities, the fusion process enables each classifier to leverage the strengths of others, compensating for potential weaknesses in individual decision boundaries. In particular, one classifier may correctly predict a class that others misclassify, allowing the ensemble to yield a more reliable final prediction.

We performed soft voting across all pairwise combinations of classifiers (SVM+MLP-A, SVM+RF, and MLP-A+RF), as well as the full trio (SVM+MLP-A+RF). This process was repeated for each of the four concatenated feature sets: V2-S + V2-M, V2-S + V2-L, V2-M + V2-L, and V2-S + V2-M + V2-L. The resulting accuracy scores are reported in Table 7.

The experimental results collectively highlight the effectiveness of both feature-level and decision-level fusion strategies in improving classification accuracy for sperm morphology analysis. Among the individual classifiers, RF achieved the highest performance when applied to the full concatenated feature set (V2-S + V2-M + V2-L), reaching an accuracy of 66.64%. The MLP-A classifier showed particular benefits from reduced feature representations, demonstrating that attention-based models can maintain, or even improve, performance while offering computational efficiency.

When decisions from multiple classifiers were fused, additional performance gains were observed. The highest accuracy (67.36%) was achieved using decision-level fusion of SVM, MLP-A, and RF over the triple-concatenated feature configuration, confirming that combining complementary classifier outputs can further enhance model robustness. Importantly, although fusion methods generally improved overall accuracy, SVM proved more effective in handling low-sample classes, emphasizing the need to consider class distribution when selecting classification strategies.

Overall, the results underscore the advantages of multi-level fusion—both at the feature and decision level—and support the proposed framework as a strong candidate for reliable, interpretable, and efficient sperm morphology classification in imbalanced datasets.

### 3.7. Comparison of Feature-Based Classifiers and Decision-Level Fusion on Low-Sample Classes

Deep convolutional networks typically require a substantial number of samples per class to achieve optimal performance. However, in many real-world datasets, including Hi-LabSpermMorpho, several classes suffer from under-representation. While data augmentation is a common approach to address class imbalance, excessive reliance on synthetic samples may reduce diversity and increase the risk of overfitting.

To address this, we evaluated the impact of alternative classification strategies on low-sample classes. In particular, we focused on classes comprising less than 2.5% of the total dataset, namely *AsymmetricNeck (1.94%), DoubleHead (0.26%), DoubleTail (1.06%), LongTail (0.22%), RoundHead (1.31%)*, and *ThinNeck (1.02%)*. We compared the classification accuracy of these classes using both SVM and soft voting fusion strategies, across three original feature combinations: V2-S + V2-M, V2-S + V2-L, and V2-M + V2-L. Table 8 presents the accuracy comparisons for each low-sample class.

The analysis reveals notable classifier-specific performance behaviors in handling low-sample classes. Although soft voting consistently demonstrates superior performance in terms of overall dataset accuracy, its advantage diminishes considerably when evaluating individual low-sample classes. For example, the soft voting method notably excels in accurately classifying *DoubleHead*, *DoubleTail*, and *RoundHead* across most feature combinations. This improvement is likely attributed to the ensemble’s probabilistic aggregation capability, allowing it to leverage complementary discriminative features provided by multiple CNN models.

Conversely, the SVM classifier significantly outperforms soft voting on the *AsymmetricNeck* and *LongTail* classes across all feature combinations. This pattern suggests that the SVM’s strength lies in its margin-maximization approach, effectively handling sparse feature spaces associated with these underrepresented classes by robustly identifying clear class boundaries despite limited sample availability.

Regarding the impact of feature fusion, combining features from EfficientNet variants typically enhances class-specific classification accuracy, particularly benefiting soft voting due to increased diversity and complementary features from different CNN architectures. However, for SVM, the feature fusion does not uniformly enhance performance, indicating its sensitivity to feature redundancy and dimensionality.

These findings underline the importance of considering class-specific behaviors when selecting classifier strategies. Decision-level fusion (soft voting) generally provides a balanced trade-off and higher accuracy on certain low-sample classes, benefiting from diverse feature sources. However, classifiers like SVM can offer critical advantages in precisely separating classes where distinct decision boundaries can be learned from limited data, highlighting their continued relevance in scenarios involving significant class imbalance and sparse training data.

### 3.8. Statistical Analysis

Table 9 presents the pairwise comparisons of each model based on their 5-fold cross-validation results using the paired *t*-test. In this analysis, the individual performances of the V2-S, V2-M, and V2-L models were compared with the performance obtained through their combination via soft voting. Additionally, these models were compared with the SVM+RF+MLP-A model, which is formed by combining SVM, Random Forest, and MLP classifiers through soft voting. The table reports the mean ± standard deviation for each model, the *p*-values from the statistical tests, whether the differences are statistically significant (*p* < 0.05), and the better performing model in each pairwise comparison. The results demonstrate that the V2-S+V2-M+V2-L ensemble significantly outperforms all other models except SVM+RF+MLP-A in every comparison. On the other hand, no statistically significant differences were observed among the individual performances of the V2-S, V2-M, and V2-L models. According to the paired *t*-test results, the second-best performing model is SVM+RF+MLP-A.

## 4. Discussion

The primary goal of this study was to improve automated sperm morphology analysis by employing advanced ensemble learning techniques. Our results demonstrate significant performance improvements using feature-level and decision-level fusion strategies over individual CNN models, highlighting the benefits of integrating complementary features from multiple architectures.

The best overall classification accuracy of 67.70% was obtained using decision-level fusion (soft voting) across EfficientNetV2 Small, Medium, and Large models. This superior performance stems from effectively leveraging multiple models’ strengths, mitigating individual model biases, and achieving a robust consensus prediction. Additionally, feature concatenation significantly enhanced the discriminative capability of the classifiers by enriching the representation space with diverse, complementary semantic features from multiple CNN architectures. Notably, RF emerged as the strongest individual classifier, achieving high accuracy on the combined feature sets due to its inherent robustness to noisy features and class imbalance, making it particularly suitable for handling complex clinical datasets.

Although the accuracy differences among various reduced feature combinations (Table 6) appear marginal, these variations are not insignificant when considered in a clinical context. The minimal change in overall accuracy can be partially attributed to the curse of dimensionality, as the concatenated feature vectors obtained from multiple EfficientNetV2 variants (S, M, and L) remain high-dimensional and partially redundant, even after dimensionality reduction. Such high-dimensional spaces may limit the separation capability of classifiers, particularly when dealing with fine-grained classes.

Moreover, the Hi-LabSpermMorpho dataset comprises 18 distinct sperm morphology classes, some of which are morphologically similar and difficult to differentiate even for trained clinicians, as noted in WHO guidelines. Therefore, a minor improvement in overall accuracy may correspond to a clinically meaningful gain, especially for challenging or rare abnormal categories.

This point is further supported by the class-wise performance reported in Table 8, where it is evident that certain rare or diagnostically critical classes benefit more substantially from the fusion strategies. For example, the F1-scores of some classes show notable improvements despite limited changes in macro-accuracy. This underscores the utility of the proposed fusion techniques in enhancing model robustness and discrimination power across difficult classes.

In summary, while the self-voting fusion of reduced features does not drastically alter the overall accuracy metrics, it plays a crucial role in boosting per-class detection quality, which is of high importance in medical diagnostics involving fine morphological classifications.

**Figure 7:**
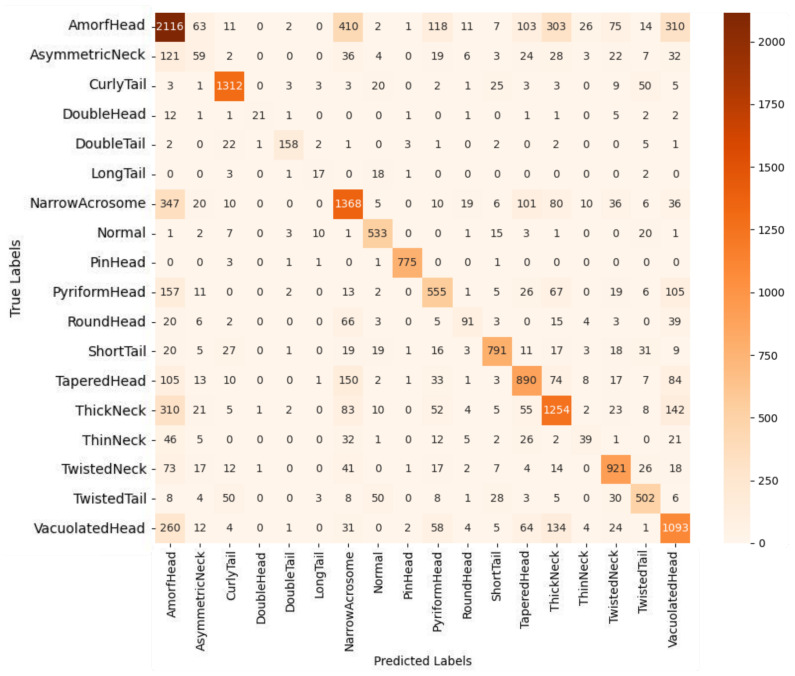
Confusion matrix of proposed ensemble model based on soft voting among EfficientNetV2-S, EfficientNetV2-M, and EfficientNetV2-L.

**Figure 8:**
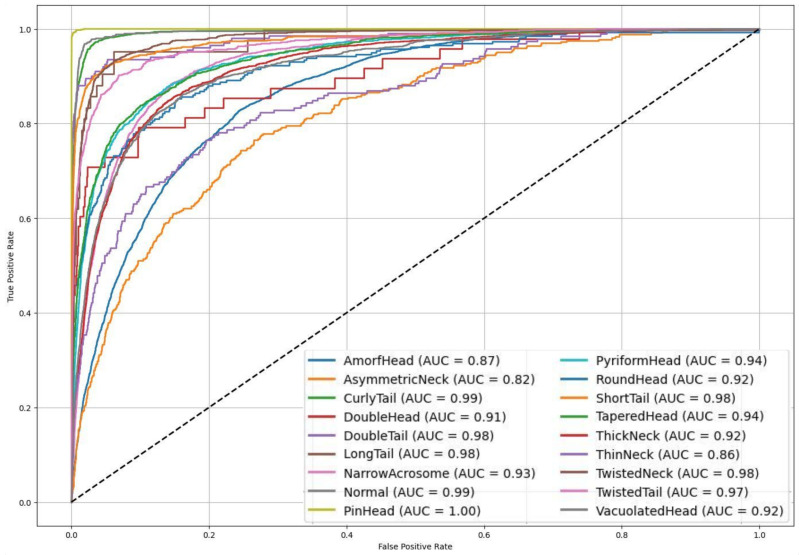
ROC curves for proposed EfficientNetV2-Small/Medium/Large soft voting ensemble model across 18 sperm morphology classes.

In addition to class-wise performance metrics, visual analyses such as the confusion matrix and ROC curves further elucidate the strengths and clinical relevance of the proposed model. In this study, the proposed multi-class deep learning-based classification model addresses the highly challenging task of identifying 18 distinct sperm morphological anomalies. As illustrated in Figure 7, the confusion matrix demonstrates high classification accuracy for morphologically distinct classes such as CurlyTail, PinHead, TwistedNeck, and TaperedHead. While some misclassifications occur among morphologically similar classes, these do not significantly impact overall performance. The overall accuracy of 67.70% is particularly promising, considering the large number of classes, data imbalance, and inherent visual complexity. Notably, the ensemble model combining EfficientNetV2 Small, Medium, and Large variants through soft voting outperformed other approaches, leveraging complementary features across scales to achieve robust and consistent classification results. Figure 8 shows the ROC curves and AUC values, further confirming the model’s strong discriminatory power, with many classes achieving AUC scores above 0.90. Moreover, a paired *t*-test conducted between the proposed ensemble model and individual CNN baselines revealed statistically significant improvements (*p* < 0.05), affirming the reliability of the observed accuracy gains beyond random variation. These findings emphasize the model’s potential as an effective and scalable solution for automated sperm morphology analysis in clinical settings.

Interestingly, while ensemble methods provided high accuracy overall, SVM demonstrated better performance for classes with fewer samples. This highlights the importance of choosing classifiers based on specific dataset characteristics, as SVM’s margin-maximization strategy effectively captures the subtle differences between sparsely represented classes.

In comparison to our prior work [21], which utilized the BesLab staining dataset and achieved a top accuracy of 65.05% with EfficientNet V2 Medium, the proposed fusion-based approach demonstrates a notable improvement. By employing ensemble learning techniques and combining CNN-derived features from multiple EfficientNet models, our method surpasses the previous best result, achieving a higher classification accuracy. This enhancement can be attributed to the use of both feature-level and decision-level fusion strategies, effectively capturing complementary information from multiple CNN architectures. Consequently, these fusion techniques substantially improve model robustness, enabling the classifier to better discriminate among the extensive and challenging 18-class sperm abnormality dataset.

Despite these advantages, our study has limitations. Although we achieved higher accuracy through ensemble methods, the dataset’s inherent imbalance remains a significant challenge, especially impacting the accuracy for minority classes. Another limitation pertains to the computational complexity involved during the training phase, as managing multiple CNN models and ensemble strategies can be resource-intensive. However, since the training is a one-time procedure and forward propagation during prediction is computationally efficient, this does not significantly restrict real-time clinical applications.

Future research directions should focus on addressing class imbalance using advanced augmentation techniques, including generative adversarial networks (GANs) to synthesize realistic minority class samples [40,41,42,43]. Additionally, exploring explainable artificial intelligence (XAI) methods, such as SHAP [44] and LIME [45], would enhance model interpretability, thereby improving clinical adoption and trust in automated systems. Finally, integrating lightweight ensemble models optimized for mobile or embedded platforms could enhance the practical applicability of these methods in clinical environments [46,47,48,49].

## 5. Conclusions

The accurate assessment of sperm morphology plays a crucial role in diagnosing male infertility and guiding assisted reproductive technologies. In this study, we proposed a robust, automated classification framework leveraging both feature-level and decision-level ensemble strategies to address the complex challenge of identifying 18 distinct sperm morphological anomalies. By fusing features extracted from multiple EfficientNetV2 variants and combining Support Vector Machines, Random Forest, and MLP-Attention classifiers via soft voting, the model achieved a notable classification accuracy of 67.36%. This accuracy corresponds to the decision-level fusion of the EfficientNetV2-S, V2-M, and V2-L models, which showed the best individual and collective performance. Meanwhile, the highest accuracy of 67.70% was obtained specifically by the soft voting ensemble of these three EfficientNetV2 variants at the feature-level fusion stage. The superiority of these ensemble configurations was confirmed by paired *t*-test analysis (*p* < 0.05), validating the statistical significance of the improvements over baseline classifiers. This performance is particularly significant considering the high intra-class variability, severe class imbalance, and morphological complexity present in the Hi-LabSpermMorpho dataset.

Our results underscore the importance of classifier selection, particularly highlighting Random Forest’s robustness and SVM’s effectiveness in minority class recognition. The use of ensemble approaches not only improved overall performance but also provided better generalization across morphologically diverse categories. Moreover, statistical analysis via paired *t*-tests confirmed the superiority of the ensemble model with significant improvements over baseline classifiers (*p* < 0.05), validating the reliability of the observed gains.

Importantly, a comparative evaluation of the ensemble strategies revealed that decision-level fusion—particularly the soft voting of EfficientNetV2-S, EfficientNetV2-M, and EfficientNetV2-L models—contributed more significantly to the overall performance improvement than feature-level fusion. This result emphasizes the advantage of combining diverse decision patterns from multiple classifiers to better handle class imbalance and subtle morphological variations.

While ensemble approaches offer substantial accuracy gains, certain limitations persist due to dataset imbalance and computational complexity. Addressing these challenges through sophisticated data augmentation, enhanced interpretability methods, and optimized computational efficiency remains crucial for future studies.

Overall, our study provides a promising step toward fully automated, scalable, and standardized sperm morphology analysis systems, with the potential to support more objective and consistent clinical decision-making in reproductive healthcare.

## Figures and Tables

**Table 1:** Hi-LabSpermMorpho dataset class distribution and number of samples.

Class Names	Number of Samples	Percentage (%)
AmorphHead	3572	19.36%
AsymmetricNeck	366	1.98%
CurlyTail	1443	7.76%
DoubleHead	48	0.26%
DoubleTail	200	1.08%
LongTail	42	0.22%
NarrowAcrosome	2054	11.13%
Normal	598	3.24%
PinHead	782	4.23%
PyriformHead	978	5.30%
RoundHead	247	1.33%
ShortTail	991	5.37%
TaperedHead	1399	7.58%
ThickNeck	1977	10.71%
ThinNeck	192	1.04%
TwistedNeck	1154	6.25%
TwistedTail	706	3.82%
VacuolatedHead	1697	9.19%

**Table 2:** Accuracies of base EfficientNetV2 models [21].

Models	Optimizers	Learning Rate	Accuracy
EfficientNetV2-S	SGD	10^−3^	65.02%
EfficientNetV2-M	SGD	10^−3^	**65.05%**
EfficientNetV2-L	RMSProp	10^−5^	64.51%

*Note.* The best accuracy is highlighted in bold.

**Table 3:** Classification accuracies for different kernels.

Models	Linear	Poly	RBF	Sigmoid
EfficientNetV2-S	62.26%	62.41%	63.08%	61.53%
EfficientNetV2-M	62.87%	63.25%	**63.94%**	62.82%
EfficientNetV2-L	63.49%	62.33%	63.81%	62.06%

*Note.* The best accuracy is highlighted in bold.

**Table 4:** Soft voting accuracy for different model combinations.

Model Combination	Soft Voting Accuracy
EfficientNetV2-S + EfficientNetV2-M	66.86%
EfficientNetV2-S + EfficientNetV2-L	66.42%
EfficientNetV2-M + EfficientNetV2-L	66.59%
EfficientNetV2-S + V2-M + V2-L	**67.70%**

*Note.* The best accuracy is highlighted in bold.

**Table 5:** Classification accuracy (%) of SVM, MLP-A, and RF on original concatenated features.

Feature Combination	SVM	MLP-A	RF
EfficientNetV2-S + EfficientNetV2-M	65.10%	64.45%	65.04%
EfficientNetV2-S + EfficientNetV2-L	65.46%	62.97%	63.94%
EfficientNetV2-M + EfficientNetV2-L	65.66%	64.11%	64.97%
EfficientNetV2-S + V2-M + V2-L	65.77%	65.93%	**66.64%**

*Note.* The best accuracy is highlighted in bold.

**Table 6:** Classification accuracy (%) of SVM, MLP-A, and RF on reduced and concatenated features.

Feature Combination of Reduced Features	SVM	MLP-A	RF
EfficientNetV2-S + EfficientNetV2-M	64.86%	65.06%	65.80%
EfficientNetV2-S + EfficientNetV2-L	64.44%	64.80%	63.7%
EfficientNetV2-M + EfficientNetV2-L	64.95%	64.99%	64.02%
EfficientNetV2-S + V2-M + V2-L	65.12%	**66.16%**	64.36%

*Note.* The best accuracy is highlighted in bold.

**Table 7:** Decision-level fusion accuracy (%) for 1280-dimensional concatenated features.

Feature Combination	SVM+MLP-A	SVM+RF	MLP-A+RF	SVM+MLP-A+RF
V2-S + V2-M	64.32%	64.77%	65.69%	65.14%
V2-S + V2-L	63.27%	63.01%	63.57%	63.31%
V2-M + V2-L	64.45%	64.92%	64.53%	64.78%
V2-S + V2-M + V2-L	66.31%	67.22%	67%	**67.36%**

*Note.* The best accuracy is highlighted in bold.

**Table 8:** Classification accuracy (%) on low-sample classes using SVM and soft voting. Each row corresponds to a different feature combination: (1) V2-S + V2-M, (2) V2-S + V2-L, and (3) V2-M + V2-L.

Classifier	Feature Combination	AsymmetricNeck	DoubleHead	DoubleTail	LongTail	RoundHead	ThinNeck
SVM	V2S + V2M	17.75%	27.08%	69.05%	16.60%	35.62%	22.39%
	V2S + V2L	12.02%	22.91%	70.50%	26.19%	27.93%	14.58%
	V2M + V2L	11.47%	25.00%	72.00%	23.80%	26.31%	12.50%
Soft Voting	V2S + V2M	8.74%	12.50%	74.00%	7.14%	34.81%	17.18%
	V2S + V2L	7.92%	33.33%	77.00%	21.42%	37.60%	18.75%
	V2M + V2L	8.74%	33.33%	76.50%	19.04%	38.46%	19.79%

**Table 9:** Statistical comparison of individual and ensemble model performances via paired *t*-test on 5-fold results.

Group-1	Group-2	Mean ± Std (Group 1)	Mean ± Std (Group 2)	*p*-Value	Reject (*p* < 0.05)	Best Model
V2-S+V2-M+V2-L	V2-S	67.70 ± 0.74	65.02 ± 0.53	0.0004	Yes	**V2-S+V2-M+V2-L**
V2-S+V2-M+V2-L	V2-M	67.70 ± 0.74	65.05 ± 0.20	0.0012	Yes	**V2-S+V2-M+V2-L**
V2-S+V2-M+V2-L	V2-L	67.70 ± 0.74	64.51 ± 0.68	0.0031	Yes	**V2-S+V2-M+V2-L**
V2-S+V2-M+V2-L	SVM+RF+MLP-A	67.70 ± 0.74	67.36 ± 0.34	0.314	No	No
V2-S	V2-M	65.02 ± 0.53	65.05 ± 0.20	0.8617	No	No
V2-S	V2-L	65.02 ± 0.53	64.51 ± 0.68	0.1721	No	No
V2-S	SVM+RF+MLP-A	65.02 ± 0.53	67.36 ± 0.34	0.008	Yes	**SVM+RF+MLP-A**
V2-M	V2-L	65.05 ± 0.20	64.51 ± 0.68	0.1024	No	No
V2-M	SVM+RF+MLP-A	65.05 ± 0.20	67.36 ± 0.34	0.0188	Yes	**SVM+RF+MLP-A**
V2-L	SVM+RF+MLP-A	64.51 ± 0.68	67.36 ± 0.34	0.0312	Yes	**SVM+RF+MLP-A**

*Note.* The best model is highlighted in bold.

## Data Availability

The datasets utilized in this work, namely Hi-LabSpermMorpho, can be found in online repositories. Repository names and accession numbers: https://github.com/Yildiz-Hi-Lab/Hi-LabSpermMorpho, accessed on 1 February 2025.
